# Increased prevalence of impulse control disorder symptoms in endocrine diseases treated with dopamine agonists: a cross-sectional study

**DOI:** 10.1007/s40618-020-01478-0

**Published:** 2020-12-12

**Authors:** G. Beccuti, F. Guaraldi, G. Natta, V. Cambria, N. Prencipe, A. Cicolin, E. Montanaro, L. Lopiano, E. Ghigo, M. Zibetti, S. Grottoli

**Affiliations:** 1 Division of Endocrinology, Diabetes and Metabolism, Department of Medical Sciences, University of Turin, Corso Dogliotti 14, 10126 Turin, Italy; 2 IRCCS Istituto Delle Scienze Neurologiche Di Bologna, Bologna, Italy; 3 Department of Biomedical and Neuromotor Sciences, University of Bologna, Bologna, Italy; 4 Division of Pediatrics, Department of Public Health and Pediatrics, University of Turin, Turin, Italy; 5 Sleep Medicine Center, Department of Neurosciences “Rita Levi Montalcini”, University of Turin, Turin, Italy; 6 Division of Neurology, Department of Neurosciences “Rita Levi Montalcini”, University of Turin, Turin, Italy

**Keywords:** Impulse control disorders, Dopamine agonists, Cabergoline, Hyperprolactinemia, Pituitary adenoma, QUIP questionnaire

## Abstract

**Introduction:**

Impulse control disorders (ICDs) have been described as a side effect of dopamine agonists (DAs) in neurological as well as endocrine conditions. Few studies have evaluated the neuropsychological effect of DAs in hyperprolactinemic patients, and these have reported a relationship between DAs and ICDs. Our objective was to screen for ICD symptoms in individuals with DA-treated endocrine conditions.

**Materials and methods:**

A cross-sectional analysis was conducted on 132 patients with pituitary disorders treated with DAs (DA exposed), as well as 58 patients with pituitary disorders and no history of DA exposure (non-DA exposed). Participants responded to the full version of the Questionnaire for Impulsive-Compulsive Disorders in Parkinson’s disease (QUIP).

**Results:**

Compared with the non-DA-exposed group, a higher prevalence of DA-exposed patients tested positive for symptoms of any ICD or related behavior (52% vs. 31%, *p* < 0.01), any ICD (46% vs. 24%, *p* < 0.01), any related behavior (31% vs. 17%, *p* < 0.05), compulsive sexual behavior (27% vs. 14%, *p* < 0.04), and punding (20% vs. 7%, *p* < 0.02) by QUIP. On univariate analysis, DA treatment was associated with a two- to threefold increased risk of any ICD or related behavior [odds ratio (OR) 2.43] and any ICD (OR 2.70). In a multivariate analysis, independent risk factors for any ICD or related behavior were DA use (adjusted OR 2.22) and age (adjusted OR 6.76). Male gender was predictive of the risk of hypersexuality (adjusted OR 3.82).

**Discussion:**

Despite the QUIP limitations, a clear sign of increased risk of ICDs emerges in individuals with DA-treated pituitary disorders. Our data contribute to the growing evidence of DA-induced ICDs in endocrine conditions.

## Introduction

Impulse control disorders (ICDs) are psychopathological conditions characterized by difficulty resisting urges to engage in behaviors that are excessive and potentially harmful to oneself or others [1]. These disorders can cause significant impairment in social and occupational functioning, as well as legal and financial difficulties [1]. The four major ICDs are pathological gambling, hypersexuality, compulsive buying/shopping, and binge eating [2].

ICDs have been described as a side effect of treatment with dopamine agonists (DAs) since 2000, when the first cases of pathological gambling were reported in patients treated for Parkinson’s disease (PD) [3]. Up to 20% of PD patients experience ICDs over the course of their illness [4]. An increased frequency of ICDs has been also described in individuals with restless leg syndrome treated with DAs [5].

Pharmacological stimulation by DAs of the D3 dopamine receptors in the mesocorticolimbic dopaminergic pathway seems to be the mechanism underlying the activation of the reward system that leads to ICDs [6].

DAs are also used to treat endocrine conditions, such as prolactin (PRL)-secreting adenoma, growth hormone (GH)- and GH/PRL-secreting adenoma, non-functioning pituitary adenoma, and idiopathic hyperprolactinemia. Therapy with DA aims to reduce levels of PRL and, in the case of adenoma, to induce tumor shrinkage. The dose of DA is generally lower for endocrine conditions (e.g., cabergoline 0.25–3.0 mg/week, up to 11 mg/week in resistant patients) than that used to treat PD [7]. Nevertheless, some cases of ICDs have been reported in prolactinomas treated with DA [8–14]. Only a few cross-sectional studies [15–17] and one prospective study [18] have evaluated the neuropsychological effect of DAs in hyperprolactinemic subjects; these studies found a relationship between DAs and ICDs.

The present study aimed to screen for ICDs and related behavior symptoms in patients with DA-treated endocrine conditions through a neuropsychological screening questionnaire.

## Materials and methods

This was an observational cross-sectional study including 180 patients enrolled in the outpatient neuroendocrine clinic of the “City of Health and Science of Turin” University Hospital (Turin, Italy) from October 2016 through February 2019. Of these, 132 patients were affected by functional hyperprolactinemia or PRL-, GH-, or GH/PRL-secreting adenoma with present or past use of DAs, defined as DA exposed; cabergoline was the only DA employed in the study. The reference group consisted of 58 patients with functional hyperprolactinemia or PRL, GH-, or GH/PRL-secreting adenoma never treated with DAs or with hypothalamic/pituitary disease without hyperprolactinemia, defined as non-DA exposed. Exclusion criteria were a history of PD and known psychiatric illness. Clinical information was collected through a review of medical records and during the survey.

All participants in the DA-exposed and non-DA-exposed groups completed the following self-administered, validated neuropsychological tools: (1) the full version of the Questionnaire for Impulsive-Compulsive Disorders in Parkinson’s disease (QUIP) to screen for symptoms of ICDs (compulsive gambling, compulsive sexual behavior, compulsive buying, compulsive eating) and related behaviors (hobbyism, punding, walkabout) [19]. The Italian QUIP version herein employed was used in an international, multicenter study on Parkinson’s disease patients [20], with the questionnaire’s last section on PD medication modified to non-PD medication. The full version assessed ICDs not only currently but also any time during DA treatment, to include in the analysis those who discontinued cabergoline. (2) Test Your Memory was used to evaluate intellectual efficiency [21] and exclude severe cognitive impairment. Lastly, (3) the validated Italian version of the Hospital Anxiety and Depression Scale (HADS) was used to assess the presence of anxiety and depressive symptoms [22].

Data are presented as medians (first and third quartiles) for skewed variables. Statistical analysis was performed using the Stata program (version 15; StataCorp LLC, College Station, TX, USA). The DA-exposed and non-DA-exposed groups were compared using the Mann–Whitney *U* test for continuous variables and using the chi-squared and Fisher tests for categorical variables.

Multivariate analysis was performed using a logistic regression model, after logarithmic transformation of all variables with a skewed distribution. A *p *value < 0.05 was considered statistically significant.

The study was approved by the local ethics committee. All patients gave written informed consent.

## Results

The population characteristics are summarized in Table 1. In the DA-exposed group, the median age was 40.5 years (27.5, 52.0) at diagnosis and 48.9 years (39.1, 62.7) at questionnaire completion; 58% were women. At study enrollment, current medications included DAs for 81% of DA-exposed individuals; 19% had previously discontinued DA treatment. Median weekly cabergoline dose was 1 mg (0.75, 1.5) and median treatment duration was 4 years (1.5, 8).

**Table 1:** Clinical characteristics of dopamine agonist-exposed and non-exposed individuals

	DA exposed (*n* = 132)	Non-DA exposed (*n* = 58)	*p *value
Gender			
Men	42%	57%	0.06
Women	58%	43%	
Age at diagnosis, years	40.5 (27.5–52.0)	52.0 (38.5–66.0)	< 0.01
Age at the time of questionnaires, years	48.9 (39.1–62.7)	66.8 (52.0–73.1)	< 0.01
Hyperprolactinemia	84%	18%	
DA treatment	Current: 81% Previous: 19%		
Cabergoline weekly dose, mg	1 (0.75–1.5); max 6.5		
DA treatment duration, years	4 (1.5–8); max 31		

Data are presented as the median (interquartile range)

DA = dopamine agonist

DA exposed = patients with pituitary disorders treated with dopamine agonist

Non-DA exposed = patients with pituitary disorders and no history of dopamine agonist exposure

Compared with the DA exposed, the non-DA exposed had a higher median age at the time of diagnosis (52.0 years; 38.5, 66.0; *p* < 0.01) and of questionnaire completion (66.8 years; 52.0, 73.1; *p* < 0.01), as well as a trend toward a lower percentage of women (43%; *p* = 0.06).

Hyperprolactinemia (PRL levels > 25 ng/mL) was observed in 84% of the DA exposed. Of these, 90% had PRL levels within a normal range after DA therapy. Normal PRL, testosterone, and both PRL and testosterone levels were found in 85%, 80%, and 76% of male DA exposed, respectively, after DA alone or in combination with testosterone replacement therapy.

In the non-DA-exposed group, only 18% of patients had hyperprolactinemia. Of these, 90% had PRL levels within a normal range, spontaneously or after neurosurgery; one subject had borderline-elevated PRL values without the need for DA treatment.

The results of the QUIP screening are summarized in Table 2.

**Table 2:** Prevalence of impulse control disorders and related behavior symptoms in dopamine agonist-exposed and non-exposed individuals

	DA exposed (*n* = 132)	Non-DA exposed (*n* = 58)	*p* value
**Impulse control disorders**			
Compulsive gambling	6%	3%	*p* = 0.36
Compulsive sexual behavior	27%	14%	*p* < 0.04
Compulsive buying	18%	9%	*p* < 0.07
Compulsive eating	22%	12%	*p* < 0.08
Positive screening for any impulse control disorder	46%	24%	*p* < 0.01 Unadjusted OR 2.70 (95% CI 1.35–5.39)
**Other related behaviors**			
Hobbyism	21%	14%	*p* = 0.17
Punding	20%	7%	*p* < 0.02
Walkabout	8%	5%	*p* = 0.53
Positive screening for any behavior	31%	17%	*p* < 0.05 Unadjusted OR 2.16 (95% CI 1.00–4.69)
Excessive amount of time spent on impulsive behaviors	7%	3%	*p* = 0.28
Difficulty in controlling the amount of time spent on impulsive behaviors	8%	5%	*p* = 0.32
Excess medication use	4%	3%	*p* = 0.92
**Impulse control disorders and related behaviors**			
Positive screening for any impulse control disorder or behavior	52%	31%	*p* < 0.01 Unadjusted OR 2.43 (95% CI 1.26–4.67)

DA = dopamine agonist

DA exposed = patients with pituitary disorders treated with dopamine agonist

Non-DA exposed = patients with pituitary disorders and no history of dopamine agonist exposure

Compared with the reference group, the DA-exposed individuals showed a higher prevalence of compulsive sexual behavior (27% vs. 14%; *p* < 0.04) and punding (20% vs. 7%; *p* < 0.02), as well as a trend toward a higher prevalence of compulsive buying (18% vs. 9%; *p* < 0.07) and compulsive eating (22% vs. 12%; *p* < 0.08), but no difference in compulsive gambling (6% vs. 3%; NS), hobbyism (21% vs. 14%; NS), walkabout (8% vs. 5%; NS), excessive amount of time spent on impulsive behaviors (7% vs. 3%; NS), difficulty in controlling the amount of time spent on impulsive behaviors (8% vs. 5%; NS), or non-PD excess therapy (4% vs. 3%; NS).

The proportion of patients screening positive for any ICD was higher in the DA-exposed compared with the non-exposed individuals (46% vs. 24%; *p* < 0.01; unadjusted OR 2.70, 95% CI 1.35–5.39), as well as for any related behavior (31% vs. 17%; *p* < 0.05, unadjusted OR 2.16, 95% CI 1.00–4.69). When considering ICDs and related behaviors together, the percentage of patients screening positive for any ICD or related behavior was 52% of the DA-exposed and 31% of the non-DA-exposed individuals (*p* < 0.01; unadjusted OR 2.43, 95% CI 1.26–4.67).

In a stratified analysis of DA-exposed men, compared with individuals not achieving normal levels of PRL and testosterone, those with a combined restoration of both hormones did not show a higher prevalence of compulsive sexual behavior (42% vs. 23%; NS), positive screening for any ICD (57% vs. 38%; NS), or positive screening for any ICD or related behavior (62% vs. 46%; NS).

Several models of logistic regression were used to identify predictors of any ICD or related behavior (Table 3a). After adjusting for gender, screening positivity was associated with DA treatment (*β* 0.37; adjusted OR 2.1, 95% CI 1.06–4.26) and age as a continuous variable (*β* 0.03; per change in regressor over the entire range: adjusted OR 6.65, 95% CI 1.58–30.06). The association remained significant after adjusting for the HADS depression score. In an unadjusted analysis, screening positivity was associated with DA duration but not dose; however, this association did not remain significant after adjusting for age and gender.

**Table 3:** Logistic regression models

Predictor	Beta	*p *value	
**(a) Predicted variable: positive screening for any impulse control disorder or related behavior**			
*Model 1* (*n* = 190)			
Gender (women vs. men)	0.31	< 0.06	
Age	0.03	< 0.02	Per change in regressor over the entire range: Adjusted OR 6.65 (95% CI 1.58–30.06)
DA treatment (no vs. yes)	0.37	< 0.04	Adjusted OR 2.10 (95% CI 1.06–4.26)
*Model 2* (*n* = 179)			
Gender (women vs. men)	0.31	< 0.08	
Age	0.03	< 0.02	Per change in regressor over the entire range: Adjusted OR 6.76 (95% CI 1.50–33.01)
DA treatment (no vs. yes)	0.40	< 0.03	Adjusted OR 2.22 (95% CI 1.10–4.26)
HADS-Depression score	− 0.04	0.40	
*Model 3* (*n* = 131)			
DA dose	− 0.02	0.87	
*Model 4* (*n* = 132)			
DA treatment duration	0.34	< 0.05	Per unit change in regressor: OR 1.40 (95% CI 1.01–1.96)
*Model 5* (*n* = 131)			
Gender (women vs. men)	0.36	0.09	
Age	0.03	< 0.03	Per change in regressor over the entire range: Adjusted OR 8.76 (95% CI 1.42–63.02)
DA dose	0.01	0.94	
DA treatment duration	0.27	0.13	
**(b) Predicted variable: positive screening for compulsive sexual behavior**			
*Model 1* (*n* = 179)			
Gender (women vs. men)	0.67	< 0.01	Adjusted OR 3.82 (95% CI 1.70–9.03)
Age	0.02	< 0.05	Per change in regressor over the entire range: Adjusted OR 6.16 (95% CI 1.03–40.37)
DA treatment (no vs. yes)	0.37	< 0.04	Adjusted OR 2.62 (95% CI 1.07–7.18)
HADS-depression score	0.07	0.44	

*OR* odds ratio, *DA *dopamine agonist

Compulsive sexual behavior was independently associated with DA treatment (*β* 0.48; adjusted OR 2.62, 95% CI 1.07–7.18), male gender (*β* 0.67; adjusted OR 3.82, 95% CI 1.71–9.03), and age as a continuous variable (*β* 0.02; per change in regressor over the entire range: adjusted OR 6.16, 95% CI 1.03–40.37), after adjusting for the HADS depression score (Table 3b).

## Discussion

This study revealed that DA treatment for endocrine conditions is associated with a higher risk of testing positive for ICDs by QUIP.

Aside from case series published in the literature [8–14], only a few endocrinological trials have investigated the relationship between DA and ICDs.

The first cross-sectional study conducted in the United States by Bancos et al. included 77 patients with prolactinomas treated with DA and 70 patients with DA-naïve, non-functioning pituitary adenomas [15]. The use of DA was associated with an increased rate of hypersexuality but not with the overall prevalence of ICDs. Interestingly, men with prolactinomas treated with DAs showed an unadjusted OR of 9.9 for any ICD compared with those with non-functioning pituitary adenomas.

A multicenter cross-sectional study included 308 patients with DA-treated prolactinoma but lacked a control group [16]. A modified QUIP showed a prevalence of 17% of any ICD; hypersexuality was most common. Independent predictive factors for ICD were male gender and alcohol use; nadir PRL did not reach statistical significance.

Another multicenter cross-sectional study enrolled 113 DA-treated hyperprolactinemic patients and 99 healthy controls [17]. Patients were more likely than controls to test positive by the QUIP-Shortened Version for any ICD, hypersexuality, compulsive buying and punding, and by the Hypersexual Behavior Inventory for hypersexuality. Independent risk factors for hypersexuality were male sex, eugonadism, lower score of the Hardy’s classification of pituitary adenomas, and psychiatric comorbidity. A higher stress score was associated with compulsive buying and punding.

A Turkish prospective study included 25 patients with prolactinomas and 63 controls (31 non-functioning pituitary adenomas and 32 healthy individuals) [18]. During a 1-year follow-up, 8% of the patients with prolactinomas developed hypersexuality, which reversed fully or decreased upon discontinuation of DA treatment.

Our study represents one of the largest samples described in the literature, enrolling patients only from a single, tertiary referral center.

Compared with the reference group, the DA-exposed patients were almost 11.5 years younger at diagnosis and 18 years younger at questionnaire completion. Because there was a trend toward a higher prevalence of women in the DA-exposed group, gender and age were used as covariates in a multiple logistic regression.

The DA-exposed individuals were more likely to test positive by QUIP for any ICD, any related behavior, any ICD or related behavior, compulsive sexual behavior, and punding. Trends toward higher rates of compulsive buying and compulsive eating were also found in the case group. No difference in the prevalence of compulsive gambling, hobbyism, walkabout, excessive amount of time spent on impulsive behaviors, difficulty in controlling the amount of time spent on impulsive behaviors, or non-PD excess therapy was found between the two groups.

Independent, positive predictors of any ICD or related behavior were DA use and age at questionnaire completion, but not male gender, which was an independent risk factor for sexual behavior only. The increased ICD risk in older age rather than younger age was not in line with previous studies of the general population, as well as PD and endocrine patients. This may be related to the clinical characteristics of our specific sample with endocrine disorders and cabergoline-induced ICDs and is, thus, not applicable in other clinical settings. The implications of these findings are still unclear, as the impact of age on the type and severity of ICD needs further investigation.

After adjusting for confounding factors, DA dose and duration did not correlate with the presence of any ICD or related behavior. The lack of interaction between DA dose and ICD risk is most likely a result of the limited statistical approach, because a resolution of ICDs has been described after DA dose reduction, not only after DA cessation [14].

To be consistent with a previous study on PD conducted by some of us [20], we planned to use the QUIP as a screening tool for ICD. This questionnaire was validated in a sample of PD patients undergoing a diagnostic interview by an investigator blinded to the QUIP results [19]. The QUIP is designed to be sensitive for the detection of ICDs and related disorders, but it is not highly specific. In fact, Weintraub et al. combined the four ICDs (compulsive gambling, compulsive sexual behavior, compulsive buying, compulsive eating) to increase the sensitivity to 97% in identifying an individual with any ICD. Similarly, they combined the ICDs with compulsive behaviors to increase the sensitivity to 96% in identifying an individual with any disorder/behavior. The negative predictive values for each ICD were very high, whereas the positive predictive values were low overall. Thus, with a high degree of certainty, a negative screen corresponds to the absence of ICD. To deal with the low positive predictive value, an individual who screens positive should undergo a clinical interview.

In our study, the high prevalence of positive screens could be supported by two hypotheses. First, the QUIP cut-off values validated by Weintraub et al. in a PD sample might not be adequate in our population, thus leading to an overestimation of the actual prevalence of ICDs. Second, the different personality profiles observed in patients with non-functioning pituitary adenoma [23] might have influenced the QUIP results in our non-DA-exposed group. It would be preferable to have the QUIP validated in an endocrine setting; the lack of a follow-up interview represents a major issue.

As a screening tool, the QUIP does not provide specific information about the severity of ICDs or related behavior symptoms. Other authors used a specific questionnaire to assess the consequences of hypersexuality [17]. This limitation suggests that a positive screen needs to be followed by a clinical interview to verify whether a patient truly has clinically significant ICDs or other compulsive behaviors and how severe they are. Another advantage of a clinical interview is to better discriminate between hypersexual diagnosis and normal return in libido. Another limitation of the QUIP is the exclusion of other impulsive activities that would fit a non-neurological clinical setting. For example, De Sousa et al. suggested the inclusion of impulsive activities like exercise, caffeine consumption, and video game use [17], and we propose mobile and social network use, trichotillomania, kleptomania, and nail biting. Also, it would be interesting to assess the ability to focus attention or concentrate, as DA-treated hyperprolactinemic patients, compared with controls, had a higher attentional impulsiveness subscale score, meaning a tendency for quicker impulsive decisions or cognitive impulsiveness [24].

Based on the QUIP characteristics, we postulate that this screening tool may be of value in detecting subclinical ICD symptoms in patients at risk for developing ICDs before starting DA treatment; however, this should be confirmed in an ad hoc study. Further studies are warranted to validate both the English and Italian QUIP in an endocrine setting, before and after DA therapy.

Aside from the questionnaire performance, a clear sign of increased risk of ICDs emerges in individuals treated with DA and appears to be consistent among PD and endocrine populations. In fact, the adjusted OR for any ICD or related behavior in our study (2.22) was similar to that described in PD (2.72) [25]. Also, an increased risk for hypersexuality was shown in our sample (adjusted OR 2.62) and in a previous study (OR 5.07 in the whole group) [15].

The gender difference in the risk of developing hypersexuality or other ICDs raises some interesting questions. As male gender is a recognized independent risk factor for ICDs [26], we speculate whether this could be due to a different neurobiological substrate between men and women, reflected in clinical disorders linked to the dopamine system. In the human brain, sex differences have been described in the extrastriatal dopamine D2 receptors of healthy individuals [27] and in the striatal dopamine D2/D3 receptor of smokers and nonsmokers [28]. Moreover, the mesostriatal and mesolimbic DA systems of rats show different sensitivities to circulating estrogens and androgens, because of specific subsets of midbrain DA neurons immunopositive for estrogen receptor β or androgen receptors. These findings provide an anatomical model with separate effects for androgens and estrogens over the mesostriatal and mesolimbic DA systems [29]. In summary, gonadal hormones appear to act like neuromodulators concurring in the sex differences in impulsive and compulsive behaviors [30].

Testosterone has been hypothesized to be permissive in the development of any ICD and hypersexuality, but no statistical association between hypersexuality and testosterone increase has been found [17]. In hypersexual men, an independent trend toward higher testosterone was found at assessment, but it did not reach statistical significance. In our study, normal PRL and testosterone levels were not associated with hypersexuality, any ICD, or any ICD or related behavior. However, our statistical power was limited, because 24% of our patients did not reach normal testosterone and PRL levels. It is noteworthy that in the literature, the relationship between testosterone and hypersexuality have been described as a trend or association that did not persist upon multivariate analysis; however, these findings may be related to limitations in the study design, the type of control group, and the lower percentage of men compared with women, which would considerably reduce statistical power.

Finally, the cross-sectional nature of the present study does not address the direction of causality. A prospective study is needed to examine any chronological relationship with the onset of ICD, because the lack of correlation between ICDs and duration of DA treatment might be due to recall bias, especially for those who had discontinued DA treatment. We also believe that the individual threshold dose for DA-induced ICDs cannot be addressed by a cross-sectional section but requires assessment in a longitudinal study.

In conclusion, we confirm that DA treatment for endocrine conditions is associated with a higher prevalence of symptoms of any ICD or related behavior and, separately, any ICD, any behavior, compulsive sexual behavior, and punding, as well as a trend toward a higher prevalence of compulsive buying and compulsive eating, as assessed by the full QUIP questionnaire.

Although lower doses of agents with low D3 receptor affinity are used in an endocrine setting, the effects are similar to those seen in PD and restless leg syndrome, confirming that ICD development is not related to a preexisting alteration of the dopaminergic system but is secondary to DA exposure.

DA use and age were predictive of the risk of any ICD or behavior, whereas male gender was predictive of the risk of hypersexuality only. DA dose and duration were not associated with ICD risk.

Although the QUIP is not validated in an endocrine setting and does not provide us with the actual frequency of ICDs as defined by standard criteria, it represents an easy-to-use, self-administered screening tool.

This large, cross-sectional study from a single, tertiary referral center contributes to the growing evidence of DA-induced ICDs in endocrine conditions.
